# Thank you to *The Lancet Regional Health - Americas*'s clinical and statistical peer reviewers in 2022

**DOI:** 10.1016/j.lana.2023.100430

**Published:** 2023-01-20

Laith Abu-Raddad

Bilal Ahmed

Ziyad Al-Aly

Graciela Alarcon

Vinicius Albani

Carlos Alencar

Warner Alpízar-Alpízar

Melania Amorim

Rebekah Amos

Katherine Anders

Evan Anderson

Jehangir J Appoo

Veronica Aran

Lars Arendt-Nielsen

Izzuddin Aris

Alejandro Arroliga

Shervin Assari

Marwan M Azar

Jorge Bacallao Gallestey

Luis Bahamondes

Ge Bai

Shasha Bai

Vicky Baillie

Amanda Baker

Mamadou Baldé

Leslie Ballas

John R Balmes

Juan Banda

Mayra Barba

Joshua Barocas

Alfred Bartolucci

Michael Barton

Francisco Bastos

Joao Bastos

Carolina Batista

Hélène Baysson

Laura E Beane-Freeman

Jonathan Bearak

Paul Beggs

Masoud Behzadifar

Melanie L Bell

Michael Bell

Jacqueline Lorene Bender

Miguel Betancourt-Cravioto

Zulfiqar Bhutta

Sagida Bibi

Katie Biello

Ilka Boin

Pablo Bolcatto

Francisco Bolumar

Stephanie Bonne

Marisa Booty

Mercy Borbor-Cordova

Sonia Borrell

Rohan Borschmann

Sarah Bott

David Boulware

Ariane Boumendil

José Carlos Bouso

Anders Boyd

Alfésio Braga

Zelinda Maria Braga Hirano

Jennifer Bragg-Gresham

Angelo Brandelli Costa

Khadijah Breathett

Bluma Brenner

Jeff Bridge

Carlos R Brites

Thomas Brothers

Ann Burchell

Mance E Buttram

Carlos Caceres

Christian Caglevic

Daniel Oliveira Cajueiro

Antônio Prates Caldeira

Elena Calvo Gonzalez

Mary Cambou

Courtney S Campbell

Jackie Campbell

Manuel Cano

Kathleen Carey

Waldemar Carlo

Suzan Carmichael

Rodrigo Carmo

Juan Caro

Adriana Carrá

Gabriel Carrasco-Escobar

Rodrigo Carrillo-Larco

Anna Cristina Carvalho

Ralph Catalano

Valeria Cavalcanti Rolla

Thiago Cerqueira-Silva‬‬‬‬

Carina Cesar

Chukiat Chaiboonsri

Esmita Charani

Yanin Chávarri-Guerra

Dimosthenis Chochlakis

Tammy Clifford

Sandra Coccuzzo Sampaio

Lara Esteves Coelho

Hugo Cogo-Moreira

Chari Cohen

Ricardo Cohen

Roger Cohen

Mercedes Colomar

Avonne Connor

Andrew Copas

Vladmir Claudio Cordeiro de Lima

Rosie Cornish

Thomas W Corringham

Maria Laura Costa

Brenda Crabtree Ramírez

Nigel Crawford

Jacob Creswell

Marcelo Crockett

Julio Croda

Cory Cronin

Diego Fernando Cuadros

Maria Curado

Sarah Cuschieri

Felicity Cutts

Leonardo Gomes da Fonseca

Sílvia Lanziotti Azevedo da Silva

Florence Dallo

Frédérick D'Aragon

Blair Darney

Claudio Dávila

Chris Davis

Nicole Davis

Aprill Dawson

Marcela de Alcantara

Everton Emanuel C de Lima

Fiorenza De Rose

Rodrigo De Toledo-Vianna

Esther de Vries

Jacobus H de Waard

Arnaud Del Bello

Oscar Del Brutto

Elaine Dennison

Ellen-Ge D Denton

Joseph Dieleman

Elizabeth Dietrich

Rhoel Dinglasan

Debora Diniz

Rajnikant K Dixit

Matthew Dodd

Carla Domingues

Rosa Maria Domingues

Brian Downer

Leszek Drabik

Chris Drakeley

Bridget Draper

Anahi Dreser Mansilla

Jan Felix Drexler

Elisabeth Carmen Duarte

Raymond M Duch

Andre Duraes

Stacie Dusetzina

Alok Kumar Dwivedi

Lucie Ecker

Benjamin Eckhardt

Erika Edwards

Katherine Ellingson

Gretchen E Ely

Marcos Espinal

Catherine Ettman

Paolo Eusebi

D Gareth Evans

Emmanuel Fadirans

Christopher Fairley

Eric Faragher

Forough Farrokhyar

Mario Fernández-Ruiz

Lucas Ferrante

Alexandre Ferraro

Carlos Ferreira

Carlos Gil Ferreira

Marcelo Ferreira

Cristiana Ferreira Alves de Brito

Elizabeth E Ferreira-Guerrero

Fernando Ferrero

Mauro Fisberg

Cynthia Fontanella

Bonne Ford

Carlos Magno Fortaleza

Marie Chantal Fortin

Anne Marie France

Jeannot Francois

Anna Freni Sterrantino

Joseph Friedman

Louisa Friedrichs

M Emmanuel Gabriel

Annette Galassi

Alison Galvani

Claudia Garcia Serpa Osorio-de-Castro

José-María García-García

Lourdes García-García

Víctor Manuel García-Guerrero

Dagmar García-Rivera

Rebecca Geary

Yenesew Gebrie

John Geyman

Alaina Giacobbe

Richard A L Gibbs

Rachel Gicquelais

Jason Gien

Mayan Gilboa

Paolo Giorgi Rossi

Davide Giuseppe Ribaldone

Peter Goldblatt

Delia Goletti

Fabio Gomes

Fabio Gomes

Héctor Gómez-Dantés

Eduardo Gotuzzo

Dylan Graetz

William Gray

Andrew Greenshaw

John Gregson

Troy Grennan

Beatriz Grinsztejn

Anneke Grobler

Eduardo Grunvald

Sarah Anne Guagliardo

Annie Haakenstad

Jessica Haberer

Sara Hackett

Samira Haddad

Pedro Hallal

Laura Hammitt

Sukwan Handali

Aaron Harris

Francis Hassard

Brett Hauber

Joanne Haviland

Yulei He

Jennifer Hellier

Linda L Henry

Ileana Heredia-Pi

Wilson Hernández

Enrique Hernández-Lemus

Elizabeth Higgs

Tarek Hijal

Andrew M Hill

Lisa Hirschhorn

Heather E Hsu

Wenbiao Hu

Anke Huels

David Hutton

Thomas Hwang

Sahra Ibrahimi

Ana-Maria Iosif

Whitney Irie

Muireann Irish

Stephanie Irving

Masako Iwanaga

Leila Janani

Omar A Jarral

Samuel Jenness

Z Gordon Jiang

Christine Johnston

David Jones

Emily J Jones

Rachel K Jones

Andrew Kaczynski

Yuko Kaneko

J'Mag Karbeah

Christiana Kartsonaki

Leila Katz

Ghazi Kayali

Richard Keijzer

Mark Kelson

Zoe Kelson

David Kershenobich

Ali Khashan

Christian Kieling

Daniel Kim

Lois Kim

Sohye Kim

Masaaki Kitajima

Jackie Kleynhans

Luke D Knibbs

Kelly Knight

Rob Knight

Rodney Knight

Jennifer Marcelle Knight-Madden

Cristian Koepfli

Kairi Kolves

Kelli Komro

Evangelos Kontopantelis

Ivan Koychev

Douglas Krakower

Noa Krawczyk

Allison Kuipers

Hannah Kuper

Hye-Young Kwon

Giuseppina La Rosa

Ronald Labonte

Marcus Lacerda

Michelle R Lacey

Darius Lakdawalla

Sham Lal

Javier Lama

Fernando Lanas

Celia Landmann Szwarcwald

Ana Langer

Peter H Langlois

Maria do Rosario Dias de Oliveira Latorre

Brian Lee

Sophie Lee

Baiying Lei

Silvana Nair Leite

Elba Lemos

Claire Leppold

Hannah Leslie

Steve Leuthner

Anna Levin

Benedikt Ley

Carmen Lim

Rodrick Lim

Bruno Lina

Mark Litaker

Xianchen Liu

Andrea Llera

Peter Lloyd-Sherlock

Stephanie Lo

Michael Long

Thomas Longden

Eduardo Luis López

Camila Lorenz

Marcelo Losso

Karen M Lounsbury

Michael Lu

Luiz Fernando Almeida Machado

Peter Macharia

Sheri Madigan

Luis Malpica Castillo

Pablo Manrique-Saide

Mohammad Ali Mansournia

Erika Manuli

Beatriz Marcheco-Teruel

Rosa Marcusso Marcusso

Ernesto Marques

Carlos Márquez

Flor Martinez Espinosa

Jesus Martinez-Barnetche

Pablo Martínez-Camblor

Kevin Martinez-Folgar

Javier Martínez-Torres

Luis Mas

Maura Massimino

Roger Mathisen

Salim Mattar

Fiona Matthews

Adam Mazurek

Elizabeth McClure

Alexander McCourt

Rosemary McEachan

Kathryn McHugh

Bryan F McNally

Elissa Meites

Melanie C Melendrez-Vallard

Andreia Melo

Cindy Mels

Jairo Mendez-Rico

Marcela Mercado-Reyes

Susan Mérillat

Robin Mesnage

Stefan Meyer

Bernardo Meza-Torres

Stefan Michiels

Christina A Mikosz

Gregorio Millett

Kara Milliron

Guadalupe Miranda-Novales

Regina Mitsuka-Breganó

Alejandro Mohar

Sergey Moiseev

Ali Mokdad

Yaser Mokhayeri

Maria Jose Monsalves

Maristela Monteiro

Fabio Moraes

Andrea Moreno

Marta Moreno

Brittany Morey

Andres Moya

Daniela Moyano

Istvan Mucsi

Samson Mukaratirwa

Edward Mulholland

Amy Mulick

Jonah Musa

Lise Musset

Cristina Mussini

Mohsen Naghavi

Gustavo J Nagy

Hiroshi Nakase

Abdorreza Naser Moghadasi

Marco Antonio Natal Vigilato

Meri Nogueira

Peter Nordström

John Norrie

Bernard North

Bernardo Nuche-Berenguer

Amy Nunn

Gabriela Oates

Nicholas Ogden

Maria Christina Oliveira

Marcelo Antônio Oliveira Santos-Veloso

Aldemir Oliveira-Filho

Abiodun Olufemi Oluyomi

Aung Oo

Jesem Orellana

Ricardo Orozco-Zavala

John Bryce Ortiz

Esteban Ortiz-Prado

Fabiola Osorio

Fundação Oswaldo Cruz

Kevin Outterson

Alisa Padon

Kathleen Page

Kimberly Page

Eduardo Dos Santos Paiva

Enny Paixao

Christopher Palmer

Diana M Papoulias

Mark Parcells

Ju Nyeong Park

Maria Parker

Jordan Parsons

Patrizio Pasqualetti

Valéria Passos

Minal Patel

Angel Paternina-Caicedo

Norma Pavía-Ruz

Clara Paz

Rebecca Pearl

Giles Peek

Stephen Pelton

Alejandro Peregrina-Lucano

Maria Peres

Julia Pescarini

Carla Pezzulo

Sophie Pilleron

Marion Piñeros

Suani T R Pinho

JoAnn Pinkerton

Laura Jean Podewils

Robert Podolsky

Jennifer Pomeranz

Luis Alberto Ponce-Soto

Marina Pontello Cristelli

Akram Pourshams

Robin Prescott

John Prindle

Catharine Prussing

Rosemary Purcell

Tina Purnat

Shan Qiao

Camille Ragin

Lauren Ralph

Anup Ramachandran

Pablo Ramirez

Otavio Ranzani

Ana Rappold

Vito W Rebecca

Peter Reese

Adrian Rendon

Paul Revill

Luis Reyes

Mitra Rezaei

Elise D Riley

Jonathan Ripp

Lucy Robertson

Rudi Rocha

Gary Rodin

Laura Rodrigues

Alfonso J Rodriguez-Morales

Ashley Roen

Modesto Rolim Neto

Camila Romano

Ethan Romero-Severson

Andrea Ronchi

Regis Rosa

Angel Rosas-Aguirre

Luis Rosero-Bixby

Jennifer Runkle

Fabian Saenz

Marc Saez

Maitreyi Sahu

Irving Salit

Zainab Samaan

Vanderson Sampaio

Claudia Sampor

Alan Sánchez

Albert Sanchez-Niubo

Fuat Hakan Saner

Susana Sans

Clemax Sant Anna

Victor Santos

Anna Satcher Johnson

Ana Sato

Andrea Schaffer

Eduardo Ekman Schenberg

Thomas Scherr

Harald Schmidt

Ione Schneider

Michael Schoenbaum

Ilan Schwartz

Gianluca Serafini

Raj Shah

Joshua Sharfstein

Affan Shoukat

Alpana Shukla

Monica Sierra

Samuel H Sigal

Denise Silva

Everton Silva

Gisele Silva

Zilda Pereira Silva

Lynn Silver

Morton Silverman

Donald Simeon

Ana Cristina Simoes e Silva

Julie Simpson

Yaser A F Snoubar

Claudia Sommer

Alonso Soto

Enrique Soto-Pérez-De-Celis

Fernando Spilki

Christoph Spinner

Sandra Springer

Allison Squires

Jeffrey Stanaway

Steffanie Strathdee

Donna Strobino

Chunlei Su

Park Su Hyun

Fernanda Surita

Magdalena Szaflarski

Emanuela Taioli

Roberto Tapia-Conyer

Cassandra Taylor

Graham Taylor

David Taylor-Robinson

João Paulo Telles

Jeff Temple

Masanori Terashima

Mishka Terplan

Alexander Testa

Ravi R Thiagarajan

Erika Thomaz

Luiz Claudio Thuler

Michael Thy

Elaine Tomasi

Tatiana Natasha Toporcov

Colm Travers

Ivan Trus

Jack Tsai

Cesar Ugarte-Gil

Michael Ungar

Diego Urrunaga-Pastor

Mary Ellen Vajravelu

Saraschandra Vallabhajosyula

Carlos Vallejos

Eric J Vallender

Antonio Carlos Vallinoto

Diederik van de Beek

Elizabeth Vaughan

Monica Vavilala

Gonzalo Vazquez-Prokopec

Saraswathi Vedam

Gustavo Velasquez-Melendez

Suzanne Veldhuis

Linda Venczel

Amanda C Venta

Gustavo Viani

Bryan Victor

Stalin Vilcarromero

Tonya L Villafana

Manuel Villaran

Andrés Villaveces

Lauren Violette

Mayumi Wakimoto

Tara Marie Watson

Fernando C Wehrmeister

Jing Wei

Daniel Weinberger

Paul Weiss

Claire Welsh

Tobias Welte

Guilheme Werneck

Parke Wilde

Annelies Wilder-Smith

Martha Slay Wingate

Tyler Winkelman

Monica H Wojcik

Roelie Wösten-van Asperen

Jie Wu

Yanyu Xiao

Yang Yang

Lixia Yao

Gwen Yeo

Darwin Yeung

Getnet Yimer

Fengqing Zhang

Jinghui Zhang

Ying Zhang

Qingguo Zhao

Carolyn Zhu

Alimuddin Zumla

